# Descriptive characteristics of continuous oximetry measurement in moderate to severe covid-19 patients

**DOI:** 10.1038/s41598-022-27342-0

**Published:** 2023-01-09

**Authors:** Jonathan A. Sobel, Jeremy Levy, Ronit Almog, Anat Reiner-Benaim, Asaf Miller, Danny Eytan, Joachim A. Behar

**Affiliations:** 1 Faculty of Biomedical Engineering, Technion, Israel Institute of Technology, Haifa, Israel; 2 Faculty of Electrical Engineering, Technion, Israel Institute of Technology, Haifa, Israel; 3 Rambam Health Care Campus, Haifa, Israel; 4 Department of Epidemiology, Biostatistics and Community Health Sciences, Faculty of Health Sciences, Ben Gurion University of the Negev Beer-Sheva, Beer-Sheva, Israel

**Keywords:** Respiration, Respiratory distress syndrome, Biomarkers, Infectious diseases

## Abstract

Non-invasive oxygen saturation (SpO2) is a central vital sign used to shape the management of COVID-19 patients. Yet, there have been no report quantitatively describing SpO2 dynamics and patterns in COVID-19 patients using continuous SpO2 recordings. We performed a retrospective observational analysis of the clinical information and 27 K hours of continuous SpO2 high-resolution (1 Hz) recordings of 367 critical and non-critical COVID-19 patients hospitalised at the Rambam Health Care Campus, Haifa, Israel. An absolute SpO2 threshold of 93% most efficiently discriminated between critical and non-critical patients, regardless of oxygen support. Oximetry-derived digital biomarker (OBMs) computed per 1 h monitoring window showed significant differences between groups, notably the cumulative time below 93% SpO2 (CT93). Patients with CT93 above 60% during the first hour of monitoring, were more likely to require oxygen support. Mechanical ventilation exhibited a strong effect on SpO2 dynamics by significantly reducing the frequency and depth of desaturations. OBMs related to periodicity and hypoxic burden were markedly affected, up to several hours before the initiation of the mechanical ventilation. In summary, OBMs, traditionally used in the field of sleep medicine research, are informative for continuous assessment of disease severity and response to respiratory support of hospitalised COVID-19 patients. In conclusion, OBMs may improve risk stratification and therapy management of critical care patients with respiratory impairment.

## Introduction

The ongoing coronavirus disease 2019 (COVID-19) pandemic spread all over the world and caused, as of early December 2021, over 5.3 million deaths. Approximately 15–20% of the confirmed cases developed severe disease, while the fatality rate was 2–5%^1–3^ depending on the country and the monitored period. In hospitalised patients with acute respiratory infections, such as patients with COVID-19, continuous non-invasive oxygen saturation (SpO2) monitoring has become central and is used to detect desaturation events required for patient triage, risk stratification and escalation of treatment, and to allow the clinicians to track responses to interventions such as oxygen enrichment or ventilation^4,5^. Specifically in COVID-19 patients, SpO2 is often used to calculate the ROX score (ratio of SpO2/FIO2 to respiratory rate) or the SpO2/FiO2 ratio to predict the need for oxygen or mechanical ventilatory support and therapy success^6–8^. These calcualtions mostly rely on admission data or discrete measurements, obtained from the hospital electronic medical record system, during hospitalization. Many models from new generation smart wearable devices such as rings, bracelets and watches include photoplethysmography sensors, that enable computation of SpO2. Moreover, several studies have demonstrated the potential of pre-symptomatic detection of COVID-19 or other viral infections from smartwatch data^9–12^. SpO2 home monitoring has successfully reduced the mortality of COVID-19 thanks to an increased hospitalization rate^13^. Thus, developing new machine learning based approaches for pre-symptomatic viral infection detection using SpO2, and other sensors, is a growing field of research^14–17^. It has not been fully elucidated how SpO2 patterns of COVID-19 patients are affected by disease severity and the level of respiratory support. Medical grade data, from hospitals’ intensive care units (ICU) and wards, will undoubtedly contribute to a better understanding of the patient’s pathophysiology. Consequently, the present work aimed to describe continous SpO2 signal characteristics along the course of the treatment of non-critical, critical with or without oxygen support and critical mechanically ventilated patients. The specific objectives of this work were to (1) identify global SpO2 signal characteristics associated with disease severity and/or the level of respiratory support, (2) determine the optimal definition of clinically relevant desaturations, (3) define SpO2 signal characteristics including desaturation parameters and OBMs that discriminate between critical and non-critical patients or those affected by the level of support, and (4) highlight the potential of oximetry derived digital biomarkers (OBMs) extracted from continuous SpO2 signals for the detection of early signs of deterioration which might require mechanical ventilation and for tracking patient responses to medical treatment. OBMs provided early signs of deterioration leading to the initiation of mechanical ventilation. The main contribution of this work was to demonstrate that OBMs can be used to monitor hospitalised COVID-19 patients efficiently and continuously.

## Methods

We describe the cohort of patients and the inclusion/exclusion criteria, and define disease severity, ventilation and oxygen support. In addition, we describe how the SpO2 signal was extracted from the bed-side monitors and how OBMs were defined. Finally, we detail how OBMs were used in a statistical framework to compare the different groups of patients (non-critical versus critical group) with or without any respiratory support (no support versus oxygen supply or mechanical ventilation).

### Study design and participants

**Figure 1:**
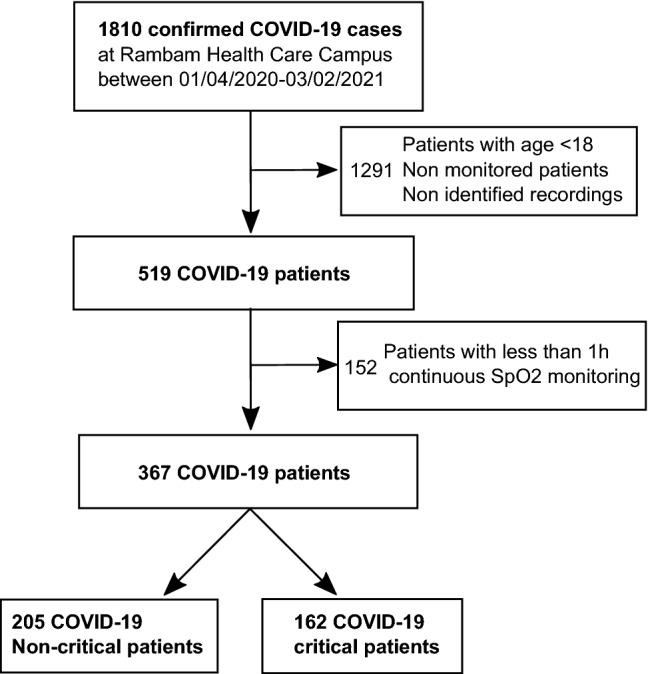
Block diagram showing the patient inclusion and exclusion criteria and groups definition.

This single center retrospective observational cohort study used electronic medical records (EMR) and continuous physiological monitoring data from Rambam Healthcare Campus (HCC), a 1000-bed tertiary academic hospital in Northern Israel. During the pandemic Rambam HCC opened five COVID-19-dedicated departments. The hospital EMR database was queried for hospitalised adult (age 18 and above) cases admitted for COVID-19, between April 1, 2020 and February 3, 2021 with at least 1 h of continuous SpO2 recording. During this period, 1810 confirmed COVID-19 cases were admitted to Rambam HCC (Fig. 1). Most cases were mild or moderate and did not necessarily involve continuous bedside monitoring. Out of the 519 adult patients monitored, 367 had more than 1 h of continuous SpO2 measurement. In total, we collected continuous measurements from 162 critical and 205 non-critical COVID-19 patients. Identified waveform data from all COVID-19 units (ward and intensive care unit, ICU) were included. Data were extracted from MINDRAY monitors (Shenzhen, China). Using the MINDRAY software CMSViewer, the available SpO2 data were exported with a resolution of 1 Hz. Overall 27 K hours of continuous SpO2 signals were collected, including 15 K hours of patients breathing room air, 4 K hours of mechanically ventilated patients (invasive respiratory support), and 8 K hours for patients under oxygen support (noninvasive oxygen support, such as mask). Patient age, sex, weight, body mass index (BMI), length of hospitalization, disease severity, and mortality rates were collected from the EMR. In addition, monitor information such as the end-tidal CO2 (EtCO2) channel for mechanical ventilation, parameters and timestamps, oxygen support and respiratory information including oxygen flow rate, and respiratory rate were collected. Comorbidities defined by ICD-9 codes were collected and analysed as in Reiner-Benaim et al^18^. Ethical approval and waiver of informed consent for this research was provided by the local institutional review board of Rambam HCC (IRB #0141–20). All methods were performed in accordance with the relevant guidelines and regulations of the Ministry of health of Israel.

### Signal preprocessing

Preprocessing of the raw SpO2 signal was performed using a block filter^19^^,^^20^ followed by a smoothing moving median filter with a window of 9 s. Raw oximetry data is often associated with missing values and artefacts caused, for example, by motion of the oximeter or lack of proper contact between the finger and the probe. The block filter discards small blocks of data with artifacts.

#### Definition of COVID-19 and disease severity

As in Reiner-Benaim et al.^18^, and as per existing guidelines^21–23^, COVID-19 positivity was defined as follow: at least one positive reverse transcription polymerase chain reaction (RT-PCR) test for SARS- CoV-2 in nasopharyngeal swab. Critically ill patients were defined as those who either received mechanical ventilation support, were hospitalised in an ICU, or were administered vasopressors (Noradrenaline or Vasopressin) or inotropes (Dopamine, Dobutamine, Milrinone, or Adrenaline).

### Oxygen and mechanical ventilatory support

Oxygen support intervals were defined based on the first and the last continuous value of oxygen flow rate extracted from the EMR for an individual patient (Figure S1). Mechanical ventilation intervals were detected using the end-tidal CO2 (EtCO2) channel from the monitors. The EtCO2, which measures the partial pressure of CO2 at end expirium, is recorded only in mechanically ventilated patients. The SpO2 data were split according to an overlap with these predefined intervals. Oxygen delivery and ventilation modes were recorded, including continuous positive airway pressure (CPAP), and bi-level positive airway pressure (BIPAP), as well as mask, nasal prongs, T-tube and tracheostomy mask use. Several devices were used, notably ventilators such as Hamilton (Hamilton Medical, Bonaduz, Switzerland), ServoAIR, Servo I (Getinge, Gothenburg, Sweden), EVITA (Dräger,Lübeck, Germany), VELA (VYAIRE MEDICAL INC., Chicago, Illinois, United States) and high flow oxygen delivery devices such as Airvo2 (Fisher and Paykel, Auckland, New Zealand) and Vapotherm (Vapotherm Inc., Exeter, New Hampshire, United States).

### Oximetry biomarkers (OBMs)

OBMs were extracted using 1 h windows of the raw SpO2 signal with a sampling frequency of 1 Hz. OBMs definitions are presented in Table S1 and were previously described by Levy et al.^24^. These biomarkers are divided into 5 categories: (1) General statistics: time-based statistics describing the SpO2 data distribution, (2) Complexity: quantifies the presence of long-range correlations in non-stationary signal, (3) Periodicity: quantifies consecutive events to identify periodicity in the SpO2 signal, (4) Desaturations: time-based descriptive measures of the desaturation patterns occurring throughout the signal, and (5) Hypoxic burden: time-based measures quantifying the overall degree of hypoxemia imposed on the heart and other organs during the recording period.

### Statistical analysis

#### Cohort

A thorough analysis of comorbidities, demographics and mortality rate was performed to characterize predispositions for both the critical and non-critical groups. Demographic variables and comorbidity rates were compared between critical and non-critical groups using the Chi-squared test or Fisher’s exact test for categorical variables, and t-tests or Mann–Whitney test for continuous variables. The p-values across all tests were corrected to control the false discovery rate (FDR) criterion^25^. Medians and inter-quartile range (IQR) were used to describe the continuous variables.

#### SpO2 global characteristics

The SpO2 signal was profiled using a density of SpO2 for each group (non-critical/critical, with or without oxygen or ventilatory support). In addition, the SpO2 density was computed per patient and support interval and was represented in a heatmap sorted by the SpO2 mean.

#### Desaturation characteristics

In order to decide which desaturation definition was the most clinically relevant, the hypoxic burden was analysed as a function of each threshold. The hypoxic burden was defined as the sum of areas of desaturations per hours for a given patient. The characteristics of the desaturation between each group were compared using the Wilcoxon test.

#### OBMs comparisons across severity and support level

The OBM toolbox was used to extract OBMs from the SpO2 signal using consecutive and non-overlapping windows of 1 h. The average of each OBM per patient and under each support type was computed for analysis. Pairwise OBMs distributions (non-critical/critical, with or without oxygen or ventilatory support) were compared using using the Wilcoxon test.

#### Time to oxygen support event analysis

Kaplan–Meier analysis^26^ was performed, where admission was taken as a start point and oxygen support initiation as a clinical endpoint. Patients who did not need support or with missing support information were censored after 30 days. Patient records lacking data recorded before the initiation of oxygen support were discarded. The support-free first hour of SpO2 recorded was considered to determine the median and the cumulative time (in percent) below 93% (CT93). Patients were divided into groups, according to their SpO2 median or CT93 in the first recorded hour. For SpO2 median, we considered the groups:(i) those with a SpO2 below or equal to 90%, (ii) those with SpO2 above 90% and below or equal 93%, and (iii) those above to 93% SpO2. For the CT93, we considered three groups: (i) below 30%, (ii) between 30% and 60% and (iii) above 60%. Cox proportional hazard models^27^ were fitted to assess the effect of SpO2 on 30-day oxygen support-free illness, with adjustment for age and sex. Kaplan–Meier analysis was performed to obtain support-free curves. The R software^28^ was used for statistical analysis. A significance threshold of 0.05 was used. Additional details on the definition of events and ventilation support used in this work can be found in the supplementary methods and supplementary Figures S1 and S2.

## Results

### Severe COVID-19 patients and hospitalisation course

Patients were split into two groups based on disease severity with 162 critical and 205 non-critical patients (Table 1). Male patients were prevalent in both groups with 60.5% in the non-critical group and 72.8% in the critical group. The in-hospital mortality was 38.9% in the critical group. The age distribution was significantly different between groups (*p* value < 0.05) with more patients 65–74 years old in the critical group and fewer patients aged 18–44 years with respect to the non-critical group. The BMI distribution was similar between groups. The length of stay (Table 2, Figure S3) was significantly longer in the critical group (*p* value < 0.001). Patients in the critical groups had significantly more overall comorbidities (*p* value < 0.01, Table 1, Table S2). Regarding vital signs at admission (Table 2), critical patients depicted a significantly lower SpO2_Room_ (SpO2 measured at breathing room air, *p* value < 0.001) and SpO2_O2Support_ (SpO2 measured under oxygen support, *p* value < 0.05) as compared to non-critical patients. The respiratory rate was significantly higher (*p* value < 0.001) in the critical group suggesting tachypnea.

**Table 1:** Population sample characteristics. BMI: body mass index.

variable		Non-critical		Critical		*p* value
		(*n* = 205)		(*n* = 162)		
sex	Male	124	(60.5%)	118	(72.8%)	0.07
	Female	81	(39.5%)	44	(27.2%)	
In-hospital mortality	FALSE	205	(100%)	99	(61.1%)	6.40E-21
	TRUE	0	(0%)	63	(38.9%)	
age group	18–44	28	(13.7%)	8	(4.9%)	0.02
	45–54	24	(11.7%)	28	(17.3%)	
	55–64	51	(24.9%)	28	(17.3%)	
	65–74	35	(17.1%)	44	(27.2%)	
	75 +	67	(32.7%)	54	(33.3%)	
BMI group	> = 20	4	(2.1%)	2	(1.3%)	0.74
	20–25	42	(21.6%)	27	(17.2%)	
	25–30	69	(35.6%)	57	(36.3%)	
	30 >	79	(40.7%)	71	(45.2%)	
Comorbidity	FALSE	67	(32.7%)	29	(17.9%)	0.01
	TRUE	138	(67.3%)	133	(82.1%)

**Table 2:** Critical and non-critical patient characteristics: demographic, length of stay, and respiratory parameters at admission.

Variable	Group	n	Mean	Median	Q1	Q3	IQR	Std	FDR adjusted *p* value	Test
Age [year]	Non-critical	205	63.05	64	52.08	76	23.92	17.17	0.084	t-test
	critical	162	66.81	68.87	55.64	77.83	22.18	14.54		
Length of stay [day]	Non-critical	205	6.72	5	3	8	5	6.73	8.50E-26	Mann–Whitney test
	Critical	162	18.17	14	8.25	21.75	13.5	15.37		
Weight [Kg]	Non-critical	194	84.37	82	72	95	23	20.24	0.2	t-test
	Critical	157	87.96	88	75	100	25	18.84		
BMI	Non-critical	194	29.21	28.7	25.35	32.37	7.02	5.18	0.28	Mann–Whitney test
	Critical	157	30.06	29.4	25.9	33.1	7.2	5.37		
SpO2_Room_ [%]	Non-critical	205	93.34	94	91	97	6	4.4	2.43E-09	Mann–Whitney test
	Critical	148	89.31	90.5	85	94.25	9.25	6.24		
SpO2_O2Support_ [%]	Non-critical	145	95.97	96	95	98	3	1.96	0.03	Mann–Whitney test
	Critical	157	94.67	95	93	97	4	3.83		
Breaths number	Non-critical	182	18.29	18	15	20	5	4.76	3.03E-07	Mann–Whitney test
	Critical	162	21.59	20	18	24.75	6.75	6.56		

### Qualitative analysis of SpO2 characteristics

**Figure 2:**
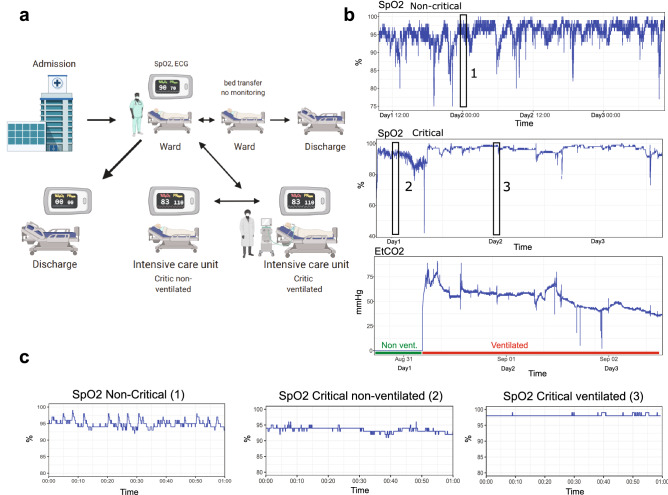
Patient in-hospital movement and examples of SpO2 signal in critical and non-critical cases. (**a**) Frequent in-hospital movement of ward monitored patients. For non-severe patients monitoring is only performed part time and a patient might be transferred or discharged depending on his health status. Image produced with BioRender.com. (**b**) SpO2 and EtCO2 signals of a non-critical and a critical COVID-19 patient with an initiation of ventilation. The transition between non-ventilated and ventilated state is clearly visible after an event of intense desaturation. Non-mechanically ventilated area is indicated in green and mechanically ventilated area is indicated in red, on the EtCO2 channel. (**c**) SpO2 signal of the same patients zoomed in on 1 h intervals, highlighted by black rectangles in panel (**b**).

A typical hospital course of a patient after admission is represented in Fig. 2A. hospitalised patients were monitored in the ward or ICU. In a non-critical case, the continuous monitoring of SpO2 of a single patient depicted frequent small desaturations, while a critical patient presented prolonged events with low SpO2 (Fig. 2B). The initiation of mechanical ventilation was visible on the EtCO2 channel. The dynamics of the SpO2 signal was noticeably impacted by mechanical ventilation, with a higher SpO2 level and reduced variability (Fig. 2C). Quantitative analysis of SpO2 characteristics.

### SpO2 distribution

**Figure 3:**
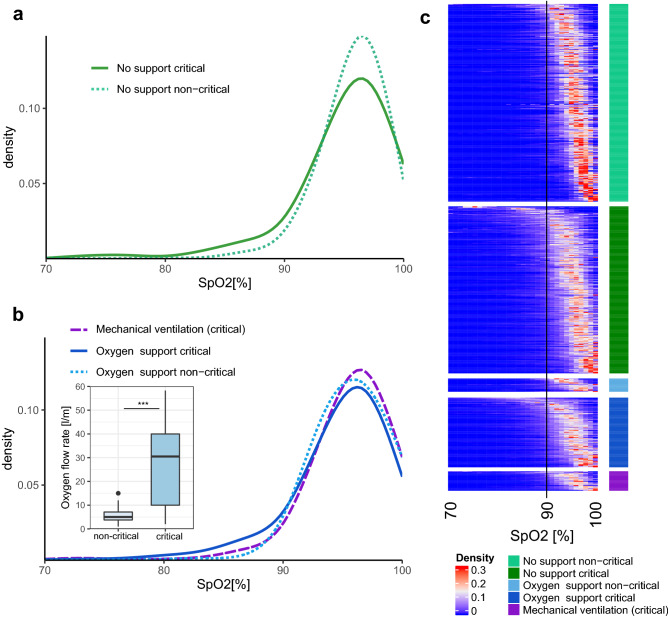
Global characteristics of SpO2 signal. (**a**) Density distribution of SpO2 for non-critical and critical patients without support. (**b**) Density distribution of SpO2 from non-critical and critical patients for intervals under oxygen support or mechanical ventilation (see Figure S1 and oxygen and mechanical ventilatory support section). The level of oxygen support (oxygen flow rate) was compared between non-critical and critical patients. The center of boxplot indicates the median, and the bottom and top edges indicate the 25th and 75th percentiles, points indicate outliers. (**c**) SpO2 density heatmap for each interval of SpO2 signal of patient in each group. A vertical black line represents the threshold of 90% SpO2 recommended in the WHO guidelines^5^ to identify severe patients.

The 95% confidence interval of continuous SpO2 measurements in non-critical COVID-19 patients was between 92 and 98% and in critical patients between 88 and 98%. The mean SpO2 was significantly lower among critical patients without support, compared to the non-critical patients (*p* value < 0.05, Fig. 3A,C). The lower SpO2 range between 80 and 90% depicted a higher density in the critical group (*p* value < 0.01). The non-critical group showed a narrower peak of density centered around 96% SpO2. A similar, although milder trend was observed in the critical versus non-critical patients under oxygen therapy (Fig. 3B,C). The median oxygen flow rate used for the oxygen support was significantly higher in critical patients (*p* value < 0.001, 3B). The use of mechanical ventilation reduced the density of low SpO2 (80–90%) to a level closer to that seen in non-critical patients. On the other hand, critically ill patients who were mechanically ventilated or on oxygen support depicted a higher density of SpO2 level between 97 and 100% compared to critical non ventilated and non-critical patients. The SpO2 density per patient in each group revealed a variety of SpO2 patterns (3C), notably in non-critical and critical patients without support. In addition, this analysis identified a sub-group of critical patients on oxygen support that did not respond well to the therapy.

### Desaturation analysis

**Figure 4:**
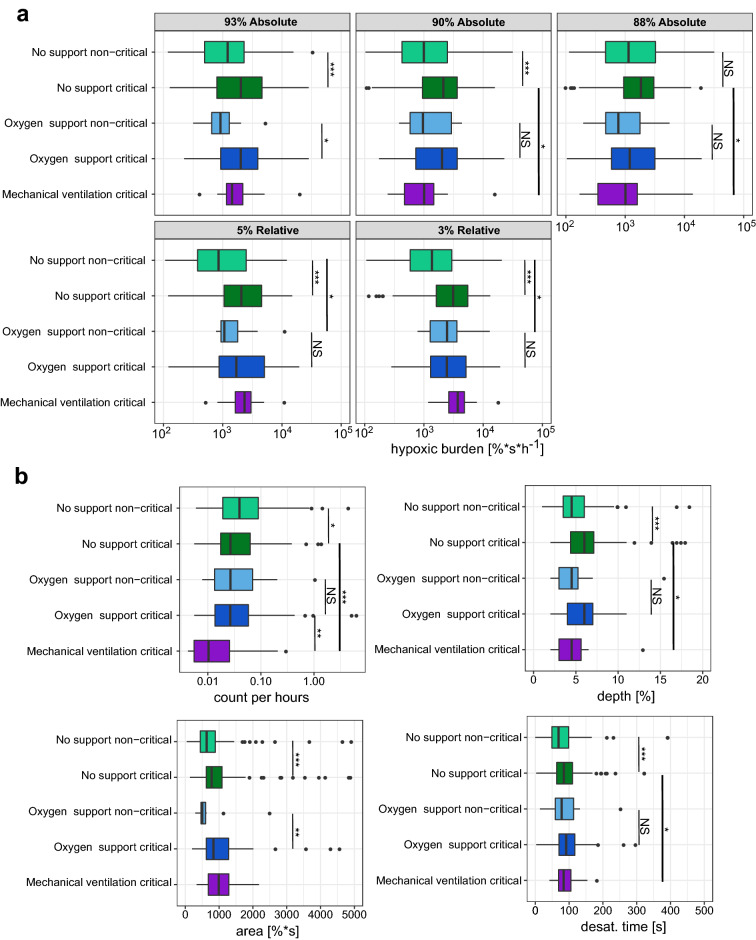
Desaturation definition and parameters. (**a**) Hypoxic burden for each relative or absolute desaturation definition (see supplementary information and Figure S2). The hypoxic burden was defined as the sum of desaturation areas normalised by recording duration. The hypoxic burden was based on relative or absolute threshold and was measured under oxygen support, mechanical ventilation or without support in critical and non-critical patients. (**b**) Desaturation characteristics for critical and non-critical patients with or without oxygen or mechanical support. The results are shown for an absolute desaturation threshold of 93%. The number of desaturations per hour, the depth the area and the desaturation time (duration between the beginning and the minimum) are represented for each group. *** Wilcoxon test *p* value < 0.001,** *p* value < 0.01,* *p* value < 0.05, NS non-significant. The center of boxplot indicates the median, and the low and high edges indicate the 25th and 75th percentiles, points indicate outliers.

Overall, the absolute threshold of 93% was the most discriminating between critical and non-critical groups (Fig. 4A), with *p* value < 0.001 and fold-change (FC) of 1.6 for no support and *p* value < 0.05 and 2.9 FC under oxygen support. A relative threshold of 3% SpO2 was able to discriminate critical from non-critical patients without support (*p* value < 0.001). Next, for the optimal definition (93% absolute), we compared the distribution of area, depth, and desaturation duration between the critical and the non-critical groups with or without oxygen support (Fig. 4B, Fig. S2). Interestingly, the non-critical group depicted more desaturations per hour compared to the critical groups (*p* value < 0.05, FC 1.8). This difference was abolished by oxygen support (non-significant, NS). Similarly, the depth and the "desat. time" (time between the beginning and the minimum) of the desaturations were significantly longer in the critical group when there was no support (*p* value < 0.001, FC 1.35). This effect was not significant under oxygen support (NS). The only desaturation parameter that showed a significant difference between the groups under oxygen support was the desaturation area (*p* value < 0.05, FC 1.6) consistent with the findings relating to hypoxic burden. Overall, the critical group showed a larger depth (*p* value < 0.001, 1.23 FC), area (*p* value < 0.001, 1.44 FC) and "desat. time" duration (*p* value < 0.001, 1.35 FC) with respect to the non-critical group. Oxygen support had a limited effect on the depth and the "desat. time", no significant differences between oxygen support and no-support were observed. Mechanical ventilation depicted a strong effect by significantly reducing the frequency of desaturations (*p* value < 0.001, 1.85 FC) and the depth (*p* value < 0.05, 1.21 FC).

### Effect of treatments and OBMs

**Figure 5:**
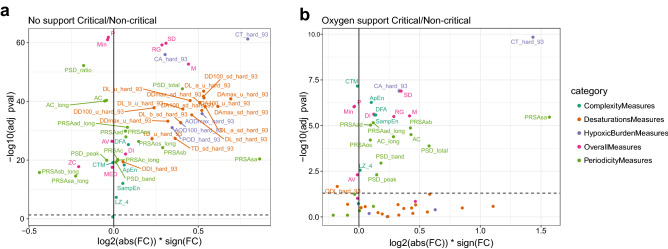
OBMs across the spectrum of disease severity and treatment support. (**a**) Critical versus non-critical without support and (**b**) critical versus non-critical under oxygen support. OBMs definition are available in Table S1. See the Figure S5 for the comparisons between no-support and oxygen support or mechanical ventilation. An OBM with a high significance (− log10 *p* value) and a large effect size (log2 Fold-change) is able to discriminate between the two groups compared in the figure. OBMs below the dashed horizontal line is insignificant. The most discriminative biomarkers could be used as indicators of patient degradation. Adj pval: FDR Adjusted *p* value, FC: Fold Change.

OBMs were investigated using a volcano plot analysis comparing between the critical and noncritical groups without support (Fig. 5A). OBMs definitions are presented in Table S1. OBMs relating to hypoxic burden and desaturation depicted a large effect (log2 fold change) with highly significant p-values (large -log10 p-values), in consistence with the results reported above. Remarkably, the integral of SpO2 < 93% (CA93) and the percentage of time < 93% SpO2 (CT93, Figure S4 ) were the most discriminating OBMs. The same analysis under oxygen support (Fig. 5B) revealed that most OBMs relating to desaturation and hypoxic burden were not significantly different between the two groups apart from CT93 and CA93. Overall, OBMs depicted reduced FDR adjusted p-values under oxygen support. However, general and periodicity related OBMs remained significantly different between non-critical and critical groups. In non-critical patients, the effect of oxygen support (oxygen support compared to no support) was mild (Figure S5A). Both FC and FDR adjusted p-values ranges were smaller than in the critical as compared to the non-critical groups (Fig. 5A). OBMs relating to hypoxic burden and desaturation parameters were still higher under oxygen support (Figure S5A). In the critical group under oxygen versus no support, the picture was similar indicating that the oxygen support had only a mild effect on OBMs (Figure S5B). On the other hand (Figure S5C), mechanical ventilation depicted a more pronounced effect on OBMs, notably with a reduction of almost all OBMs.

### Transition analysis and OBM trajectories

There were a total of 43 patients with transitions, i.e. initiation of mechanical ventilation, with a total of 68 transitions overall. Among these, there was a Q1 of 1, and a Q3 of 2 transitions, and the maximum number of transitions per patient was 3. Panel A of Figure S6 shows examples of transitions of three representative patients and panel B shows the average profile of each detected transition. Temporal tracking of selected OBMs is illustrated in Figure S7. The median SpO2 depicted a larger variation about 1 h before the transition. The desaturation class represented by the oxygen desaturation index (ODI93), depicted large variations around the transition. The ODI93 was lower number about 1.5 h before the transition suggesting larger desaturations in agreement with CA93 (hypoxic burden) which increased and showed a high variance before the transition. Approximate entropy ApEn (complexity) was slightly reduced about 4 h before the transition and markedly lower after the transition. Finally, PSDtotal (Periodicity) showed an increasing perturbation between 4 h to 1.5 h before the transition. Thus, before the transition, the spectral band of interest (0.014 − 0.033 Hz) appeared to have a greater power than the rest of the signal.

### Time to oxygen support event analysis

**Figure 6:**
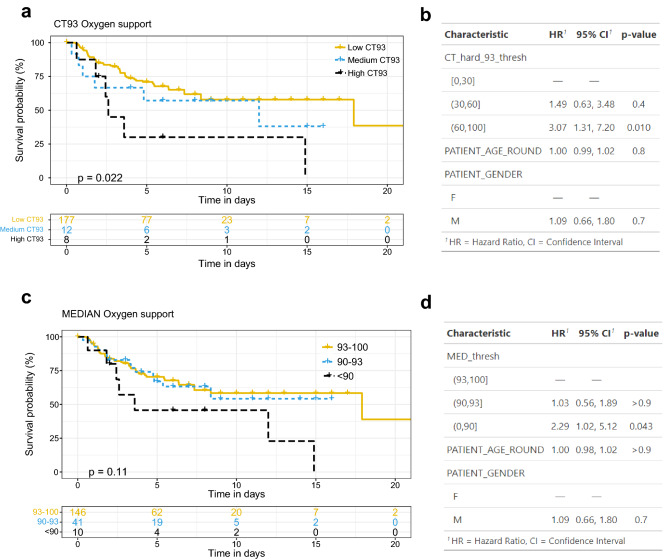
Time to event analysis of oxygen support using SpO2 CT93 and median measured during the first recorded hour. (**a**) Kaplan–Meier analysis of CT93 stratified as low (0*–*30), medium (30*–*60), and high (60*–*100) CT93. (**b**) Cox proportional hazards model summary, including stratified CT93, age, and sex. CT93 represents the fraction of time (in %) under the 93% SpO2 threshold. (**c**) Kaplan–Meier analysis of median stratified as bellow 90%, between 90 and 93% and above 93%. (**d**) Cox proportional hazards model summary, including stratified median, age, and sex.

Using OBMs extracted from the first hour of recording, we investigated whether a high CT93 significantly affected the time to event curves for the initiation of oxygen support. In other words, we wanted to test if a high CT93 resulted in a faster initiation of oxygen support. Figure 6A depicts the Kaplan–Meier analysis of patients stratified with low, medium, or high CT93. The group with high CT93 (60–100) showed an oxygen-free curve which decayed faster compared to the other groups (*p* value = 0.022), suggesting that most of these patients were rapidly supported with oxygen. A multivariate Cox model with adjustment for age and sex presented a hazard ratio (HR) of 3.07 (95% CI: 1.31–7.20, *p* value = 0.01) for the high CT93 group relative to the low CT93 group (Fig. 6B). A similar analysis with the median of the first hour of recording is shown in Fig. 6C. A slightly smaller, yet significant effect was found (HR = 2.29, 95% CI: 1.02–5.12, *p* value = 0.043, Fig. 6D).Taken together, this analysis suggested that high CT93 was more highly associated with oxygen support requirements than the median SpO2.

## Discussion

While oximetry of healthy people generally shows oxygen saturation levels in the range [94%-98%]^29^, critical COVID-19 patients showed a higher density of low SpO2 (80–90%) as compared to non-critical or critical ventilated patients (Fig. 3). Specifically, the 95% confidence intervals of SpO2 were [92–98%] and [88–98%] for non-critical and critical COVID-19 patients respectively. Thus, several questions arise: what is an appropriate SpO2 threshold to distinguish critical from non-critical patients? what is a good predictor for oxygen support and mechanical ventilation needs? How does ventilation and oxygen support affect the SpO2 level and its dynamics? Several studies have started to investigate these questions. A recent study showed that oxygen saturation below 90% is associated with a higher probability for mortality^30^, but the work was based on discrete measures of SpO2. Another recent analysis of SpO2 and the ROX scores at admission concluded that an SpO2 below 78% was a good predictor of the need for mechanical ventilation^6^ and that a ROX score above 1.4 while on non-invasive ventilation was a good predictor of support failure. Other trials suggested that the SpO2/FiO2 ratio (or PaO2/FiO2) can serve as a prognostic marker and facilitate early adjustment of treatment^7,31,32^. In the present study, we demonstrated that an oxygen saturation threshold of 93% best differentiates between critical and non-critical patients with or without oxygen support. Thus, our work suggests a more stringent threshold compared to the WHO guidelines^5^ that define an operational threshold of 90% SpO2 to define severe patients. In addition, a drop of 3% SpO2 (relative threshold) discriminated critical from non-critical patients without support. This is consistent with the recommendation for prompt assessment in the emergency oxygen therapy guidelines^33^ as it may indicate an acute deterioration in the patient’s condition. In addition, our work highlighted the differences of SpO2 dynamics between critical and non-critical patients.

Specifically, we observed that the non-critical group frequently had shallow desaturations, while critical patients had deeper and longer but less numerous desaturations (Fig. 4). Our work also found that oxygen support drastically reduced the differences of OBMs between the two groups. Mechanical ventilation was associated with a reduction in the SpO2 signal complexity and, periodicity, a lower incidence of desaturation and a higher overall SpO2 saturation level with a risk of over oxygenation which may be detrimental^34,35^. In addition, we showed that various biomarkers and standard analysis of continuous oximetry previously developed to study obstructive sleep apnea or chronic obstructive pulmonary disease^20,24,36,37^ may support the monitoring of COVID-19 patients. Strikingly, CT93 and CA93, two OBMs related to the hypoxic burden class were most discriminative between critical and non-critical patients regardless of oxygen support. In addition, our work showed that a high CT93 (above 60%) during the first hour of monitoring was more highly associated with the need for oxygen support than a median SpO2 >  = 90%. This discriminative capacity of high CA93 was consistent with a recent study suggesting cumulative oxygen deficit (based on PaO2) as a predictor of mechanical ventilation^38^. The presented work has several important limitations. First, it was a single center retrospective and descriptive study. Second, it did not explore the effect on SpO2 for sub-type of support or stratified parameters such as FiO2, EtCO2, PEEP or pressure support levels. Third, OBMs were computed from 1 h windows, limiting the capacity to capture certain patterns. The OBM analysis is based on a feature vector of 175 features. In addition, OBMs from the same OBM category with slightly different parameters might present some level of collinearity, which is not detrimental in a descriptive univariate analysis apart from affecting the FDR due to the large numbers of variables tested. In the case of a multivariate analysis, model selection algorithms should be applied.

This study bears important potential clinical implications. SpO2 monitoring has the advantage of being used frequently and continuously in all patients requiring oxygen treatment, and the data can be saved and processed. The collected OBMs may serve as a tool to predict the patient’s trajectory while being treated with oxygen supplementation or non-invasive ventilation. In addition, assessing oxygenation and desaturation patterns might serve as a prognostic tool for COVID-19 patients. This prognostic tool could be based on a machine learning classifier using our OBMs and other clinical characteristics of the patients as features. Third, in an overwhelmed medical system, the decision whether to admit or discharge a patient to a ward or ICU is extremely important. Analyzing oxygenation can assist in that manner ^4^^,^^39–42^. Furthermore, emerging wearable technologies such as smart watches, smart rings and bracelets include SpO2 sensors. The knowledge and the data generated in hospitals can be beneficial toward the development of new algorithms to enable smart home oximetry monitoring and an early alert system for hospitalisation. Finally, our results can be relevant in other medical conditions involving the respiratory system such as pulmonary infections, chronic obstructive pulmonary disease, acute respiratory distress syndrome and others. Large scale, preferably prospective randomized trials will be required to validate our results. In conclusion, this work is the first report of continuous SpO2 signal analysis in COVID-19 patients across severity categories and respiratory support levels. it demonstrated that continuous monitoring of SpO2 is of paramount importance toward characterization and management of COVID-19 patients. In addition, it showed that the oximetry signal contains a lot of untapped clinically relevant information. Mechanical ventilation and oxygen support have a striking impact on the SpO2 signal characteristics. Finally, OBMs may improve monitoring of patients and enable prediction of deterioration of their status.

### Schematic drawing

Scheme in Fig. 2a was created with the web application of BioRender.com.

## Supplementary Information

Supplementary Information.

## Data Availability

The anonymised database including the waveforms and the clinical data from the COVID-19 patients will be accessible upon reasonable request to corresponding author, which will be individually reviewed by the ethical committee of the Rambam HCC. The source code of the POBM toolbox used in this research is available at physiozoo.com and at https://oximetry-toolbox.readthedocs.io/en/latest.
