# Supplementary material for: Descriptive characteristics of continuous oximetry measurement in moderate to severe covid-19 patients
Source: Sci Rep. 2023 Jan 9;13:442. doi: 10.1038/s41598-022-27342-0 (PMC9828367; doi:10.1038/s41598-022-27342-0)
Supplement: Supplementary file 1 — Supplementary Information. [file 41598_2022_27342_MOESM1_ESM.docx]

Supplementary information of

*“Descriptive characteristics of continuous oximetry measurement in moderate to severe COVID-19 patients”*

Jonathan A. Sobel^1*^, Jeremy Levy^1,2^, Ronit Almog^3^ , Anat Reiner-Benaim^4^ , Asaf Miller^3^ , Danny Eytan^3^ and Joachim A. Behar^1^

Supplementary Information

Events definition.

Desaturation events. Two categories of desaturations can be defined: (1) "relative" desaturations corresponding to a decrease of x% (here taken as 3% or 5%) of the SpO2 signal, before returning to 1% below the initial saturation level. The relative threshold desaturation detector is based on the Oxygen Desaturation Index (ODI) which is traditionally used in sleep medicine. The ODI algorithm from Jung et al.^41^ was used with its implementation and validation by Behar et al.^42^; (2) "hard" desaturations defined as SpO2 level falling below a given threshold of x% SpO2 (here taken as 93%, 90% or 88%). When the SpO2 signal falls below this value, a desaturation is detected. Figure S2 presents examples of desaturations detected using the hard threshold and relative threshold. The event has to be of a minimum length of 120 seconds. This is to be considered a desaturation event to avoid a false positive reading caused by the patient’s movements or noise. For each detected desaturation, several desaturation parameters were extracted. Specifically, area, length, depth, time between the beginning and the minimum point (defined as desat. time), the time between the minimum and the end (defined as resat. time) and the duration between two consecutive desaturations (interval time).

Transition to mechanical ventilation event. For each patient in the critical group, the time of initiation of mechanical ventilation was extracted on the EtCO2 channel using a sliding window procedure. Briefly, two consecutive windows of five seconds long were created. When sliding the two consecutive windows over the CO2 signal, if the first one had no signal within it whereas the second one had only valid values within it, this was labeled as an event of transition to mechanical ventilation.

OBMs and transition events.

A sliding window of 30 minutes with a shift of 5 min was applied on the SpO2 signal 8h before to 4h after transitions to study the dynamic of the signal and identify candidate OBMs anticipating a deterioration of the patient requiring intubation and mechanical ventilation. The median and the interquartile range of all transitions are represented for selected OBMs that depicted large variations before the transition.

**Table S1**. Oximetry derived biomarkers (OBMs) definitions adapted from levy et al.^24^

**Table S2.** Comorbidities prevalence among study patients at admission and comparison between critical and non-critical groups.

| variable |  | non-critical | | critical |  | FDR adjusted  p-value |
| --- | --- | --- | --- | --- | --- | --- |
|  |  | (n = 205) |  | (n = 162) |  |  |
| cancer ALL | FALSE | 190 | (92.7%) | 145 | (89.5%) | 0.48 |
|  | TRUE | 15 | (7.3%) | 17 | (10.5%) |  |
| cancer SOLID | FALSE | 191 | (93.2%) | 146 | (90.1%) | 0.45 |
|  | TRUE | 14 | (6.8%) | 16 | (9.9%) |  |
| cancer HEMATOLOGIC | FALSE | 204 | (99.5%) | 160 | (98.8%) | 0.69 |
|  | TRUE | 1 | (0.5%) | 2 | (1.2%) |  |
| acute renal failure | FALSE | 199 | (97.1%) | 153 | (94.4%) | 0.4 |
|  | TRUE | 6 | (2.9%) | 9 | (5.6%) |  |
| CKD | FALSE | 183 | (89.3%) | 132 | (81.5%) | 0.14 |
|  | TRUE | 22 | (10.7%) | 30 | (18.5%) |  |
| asthma and bronchiectasis | FALSE | 197 | (96.1%) | 158 | (97.5%) | 0.68 |
|  | TRUE | 8 | (3.9%) | 4 | (2.5%) |  |
| COPD | FALSE | 195 | (95.1%) | 153 | (94.4%) | 0.81 |
|  | TRUE | 10 | (4.9%) | 9 | (5.6%) |  |
| dementia | FALSE | 201 | (98%) | 155 | (95.7%) | 0.36 |
|  | TRUE | 4 | (2%) | 7 | (4.3%) |  |
| diabetes | FALSE | 137 | (66.8%) | 93 | (57.4%) | 0.2 |
|  | TRUE | 68 | (33.2%) | 69 | (42.6%) |  |
| hypertension | FALSE | 102 | (49.8%) | 76 | (46.9%) | 0.74 |
|  | TRUE | 103 | (50.2%) | 86 | (53.1%) |  |
| hyperlipidemia | FALSE | 133 | (64.9%) | 85 | (52.5%) | 0.07 |
|  | TRUE | 72 | (35.1%) | 77 | (47.5%) |  |
| ischemic heart disease | FALSE | 178 | (86.8%) | 138 | (85.2%) | 0.78 |
|  | TRUE | 27 | (13.2%) | 24 | (14.8%) |  |
| cardiovascular disease | FALSE | 83 | (40.5%) | 52 | (32.1%) | 0.22 |
|  | TRUE | 122 | (59.5%) | 110 | (67.9%) |  |
| smoking | FALSE | 181 | (88.3%) | 140 | (86.4%) | 0.75 |
|  | TRUE | 24 | (11.7%) | 22 | (13.6%) |  |
| stroke | FALSE | 189 | (92.2%) | 143 | (88.3%) | 0.36 |
|  | TRUE | 16 | (7.8\) | 19 | (11.7%) |  |


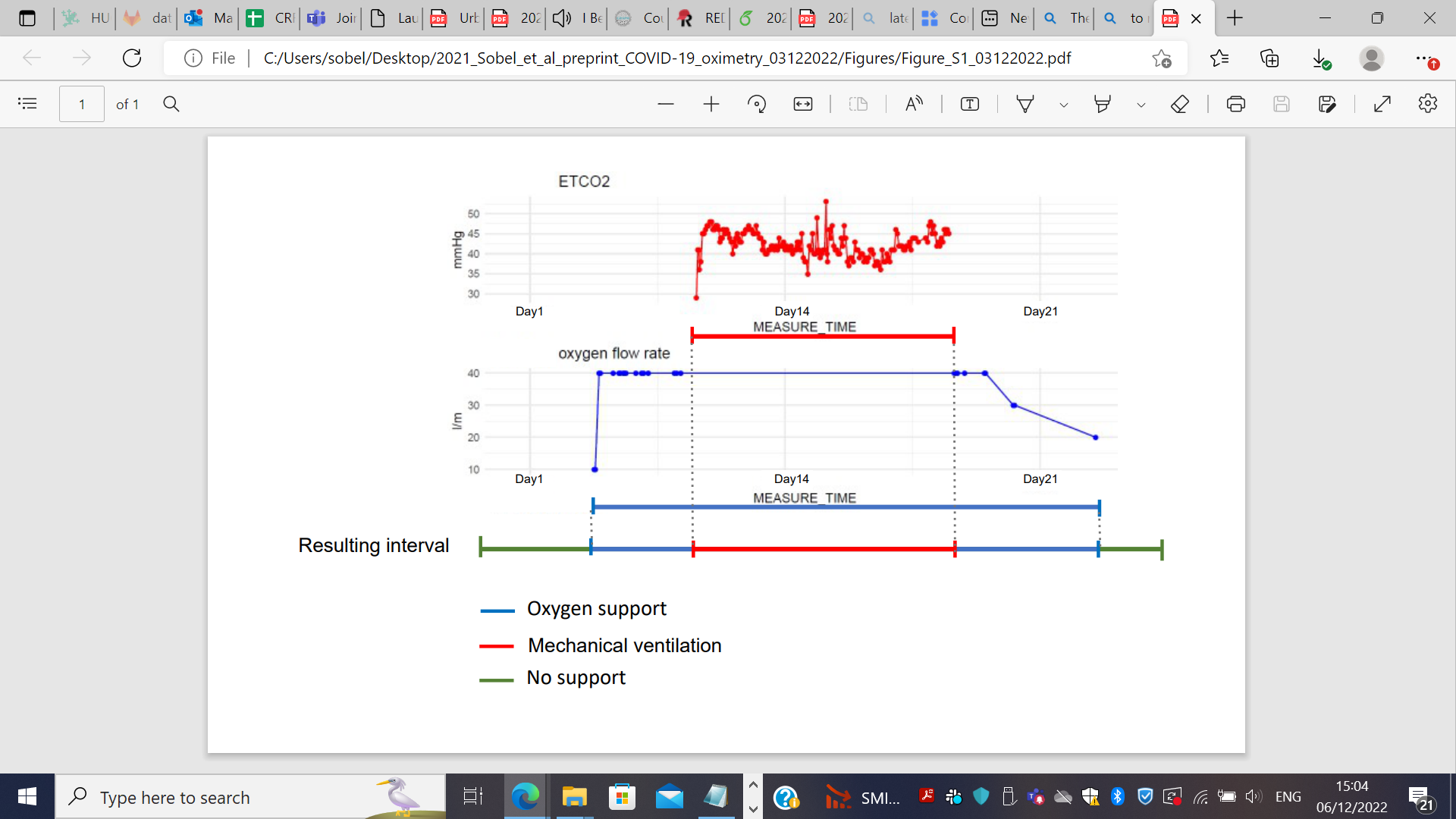


**Figure S1** Definition of oxygen support and mechanical ventilation intervals. The first and the last time stamp are used from the oxygen flow rate and from the EtCO2 channel. No support is defined between the admission and the first information about support (or the last and the discharge) or if there is no support information, the whole monitoring is considered without support.


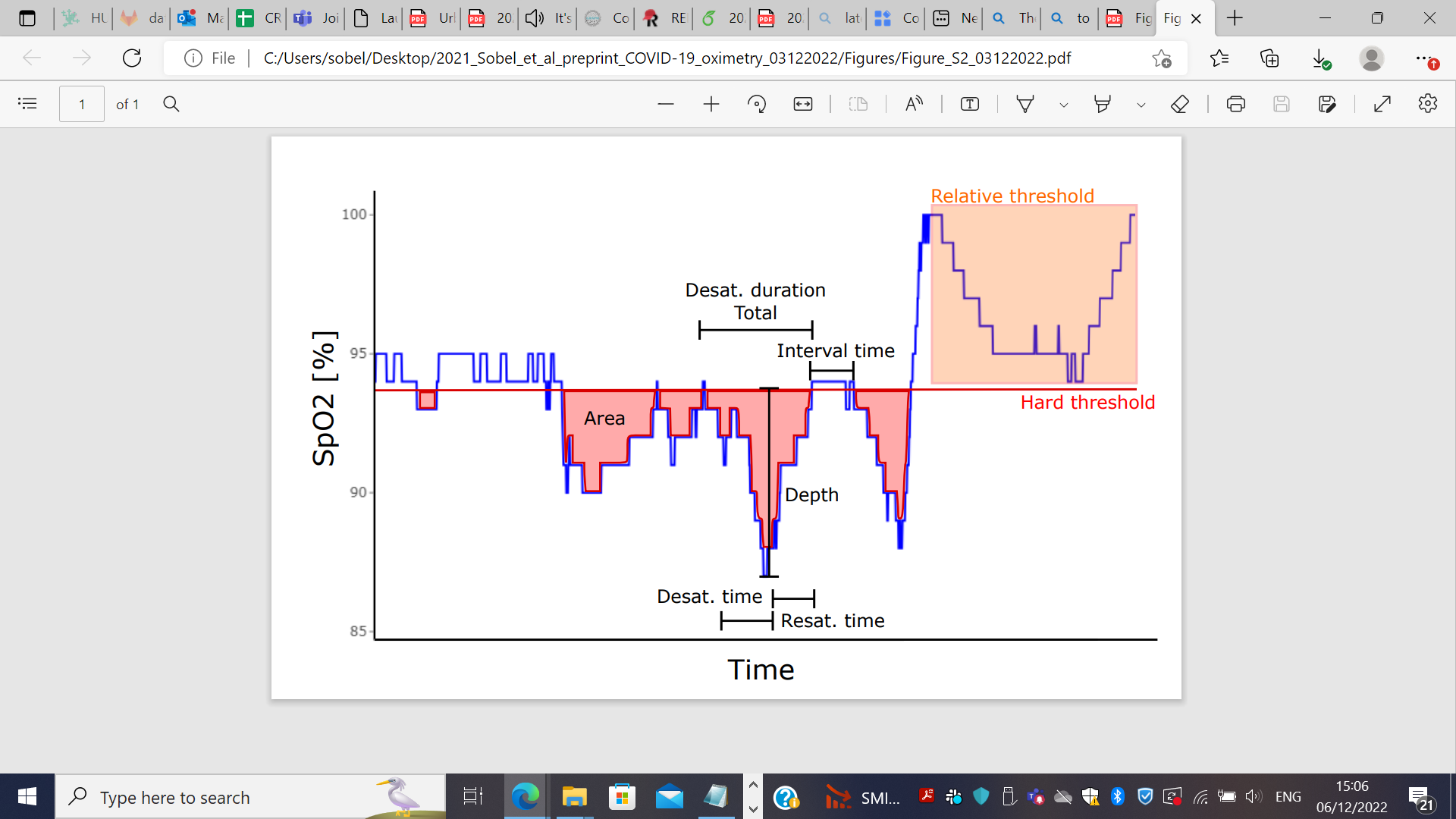


**Figure S2** Example of SpO2 signal with desaturation parameters highlighted. Here the hard threshold is at 93% and the relative threshold is of 5%.


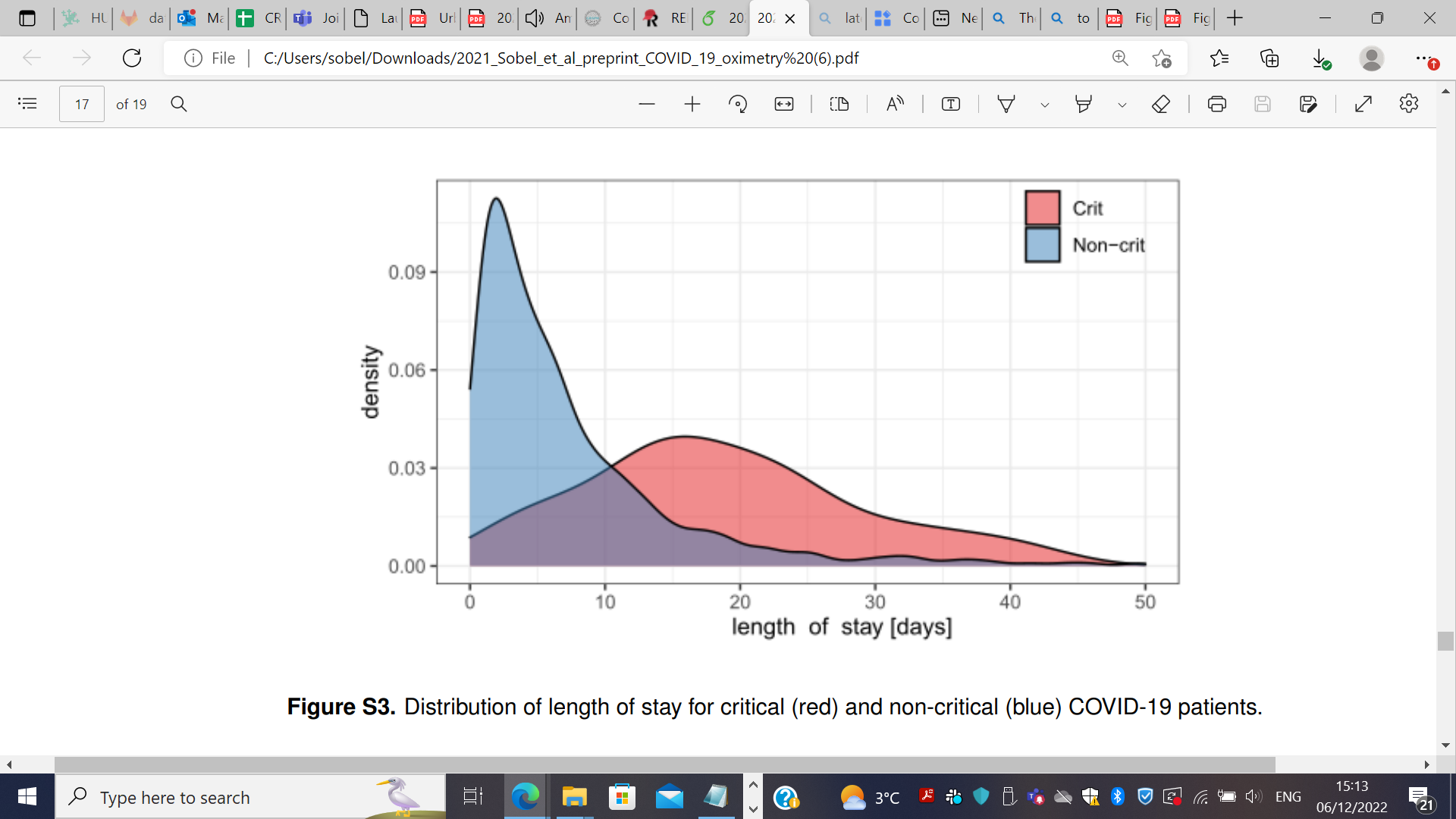


**Figure S3** Distribution of length of stay for critical (red) and non-critical (blue) COVID-19 patients.


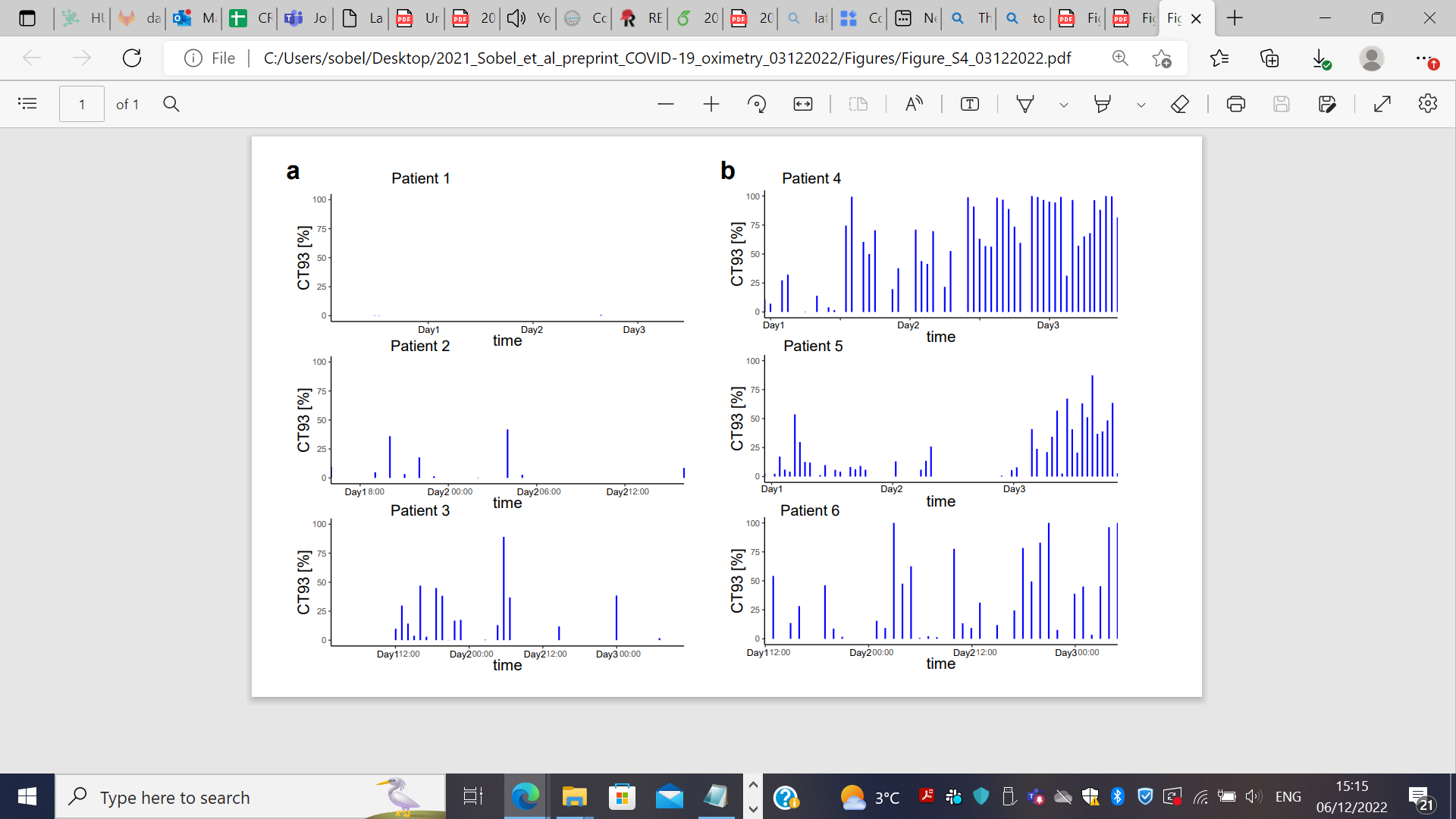


**Figure S4** OBM CT93, defined as the percentage of time under the 93% SpO2 threshold, extracted from 1h windows for representative **a**) non-critical and **b**) critical patients without support. CT93 was the most discriminating biomarker between critical and non-critical group.


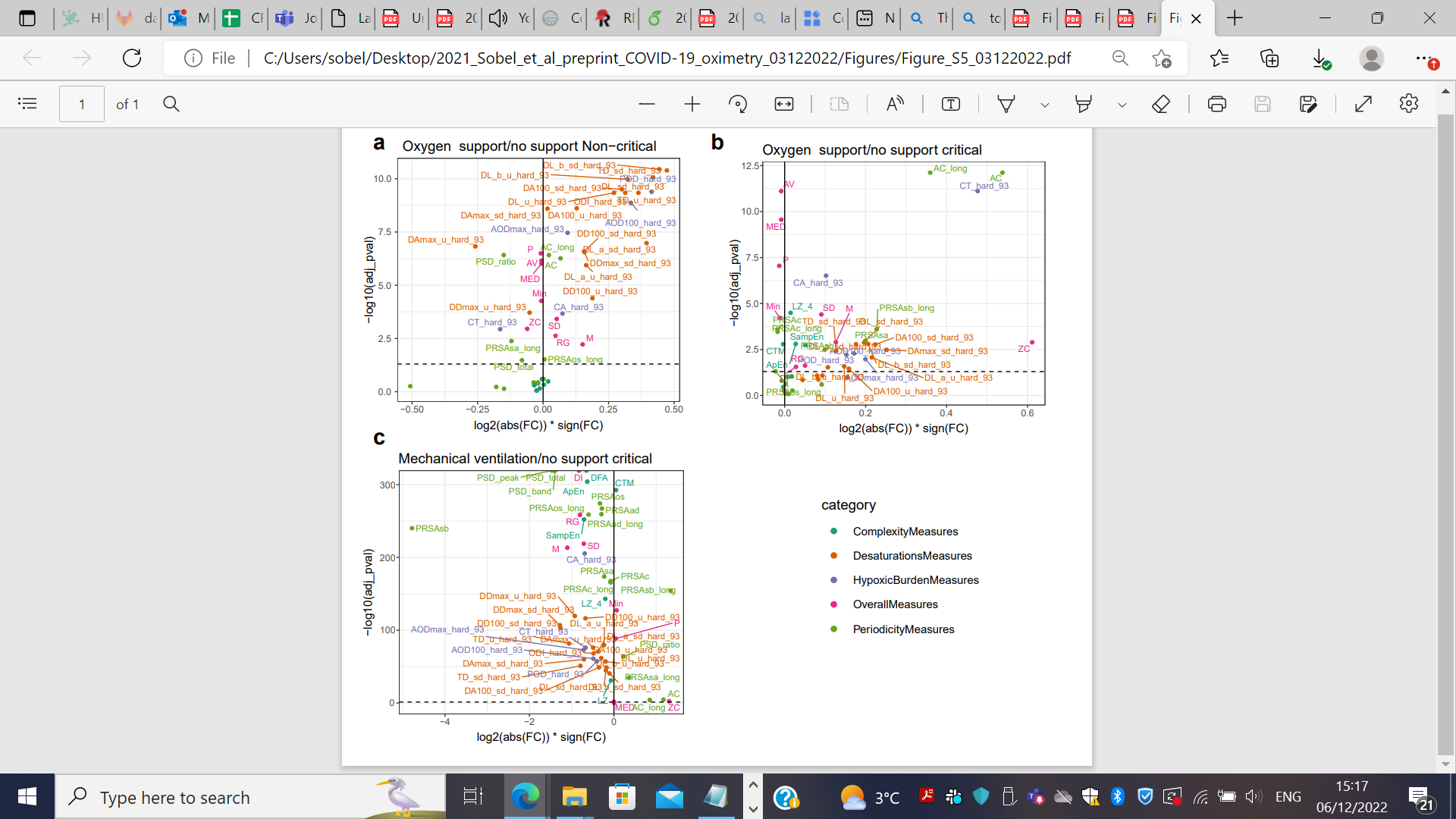


**Figure S5** OBMs across the spectrum of disease severity and treatment support. **a**) Non-critical under oxygen compared to non-critical without support. **b**) Critic under oxygen compared to critical without support. **c**) Critic under mechanical ventilation compared to critical without support. OBMs definition are available in Table S1.


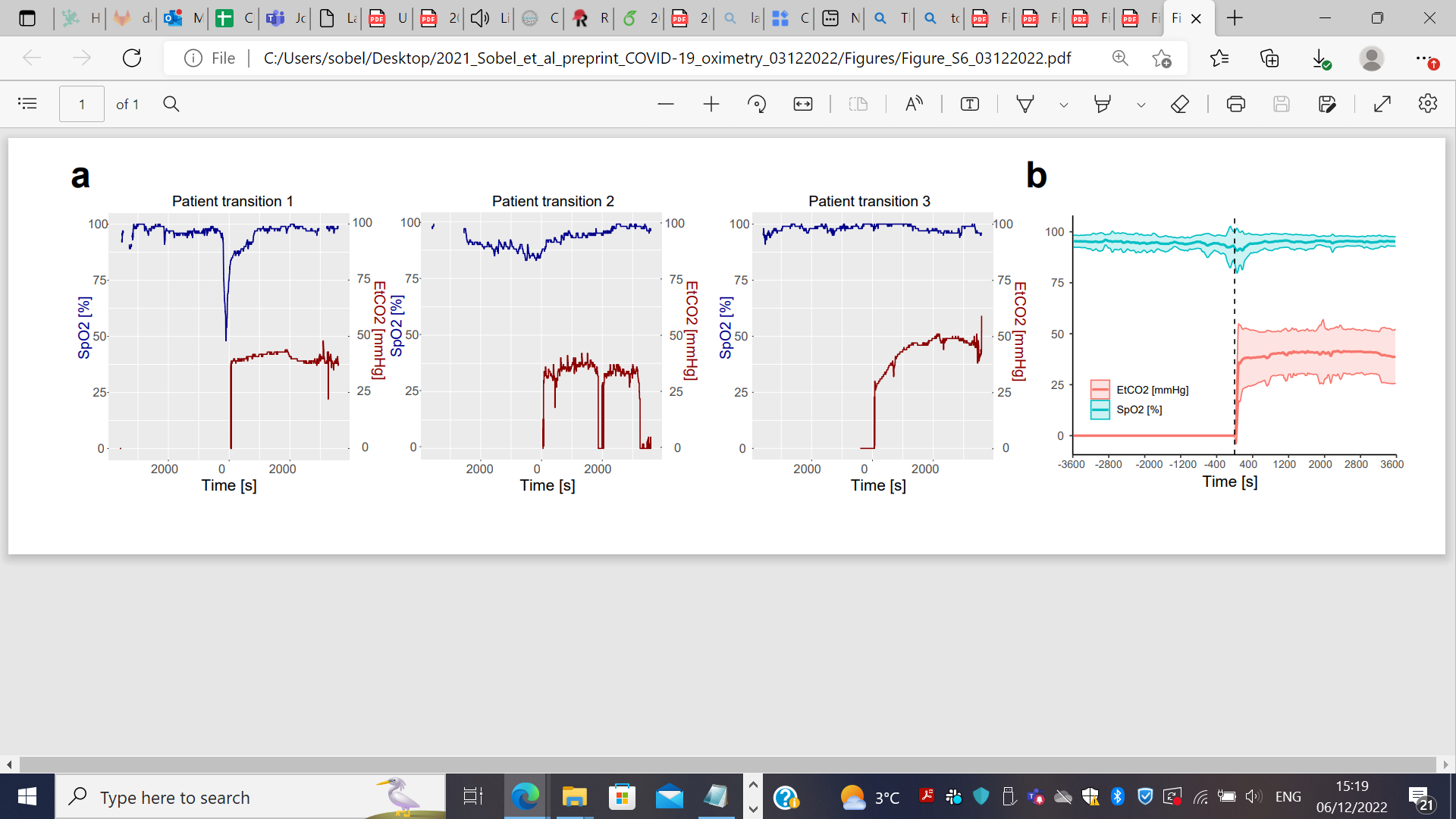


**Figure S6** Effect of the ventilation on SpO2 signal at the initiation of the ventilation. **a**) Representative initiation of ventilation in three different patients. EtCO2 (red) and SpO2 (blue) signal were extracted using 2h window centered on the ventilation initiation. **b**) Average and standard deviation of EtCO2 and SpO2 for all the detected transitions.


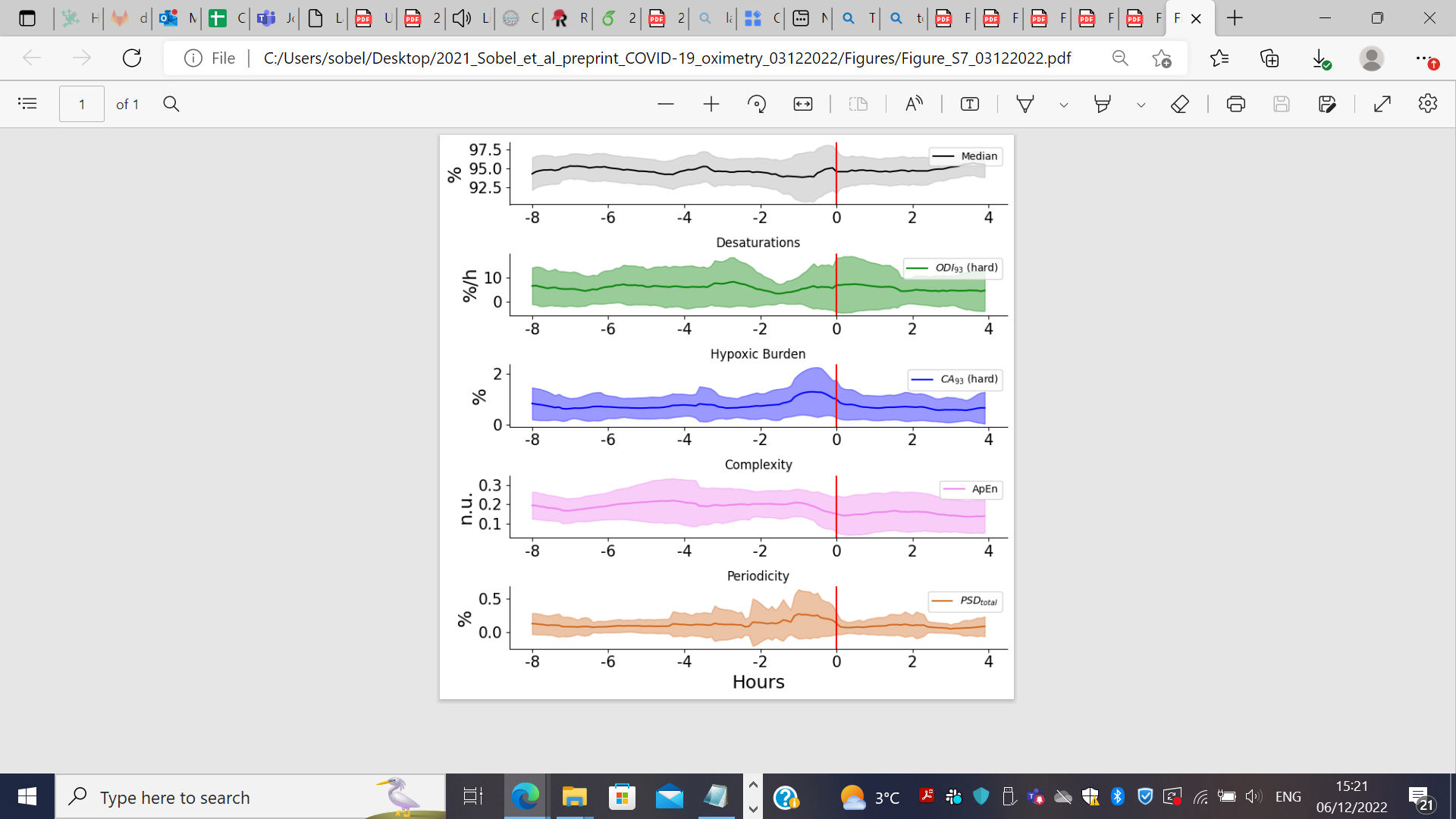


**Figure S7** Temporal tracking of OBMs before and after the initiation of mechanical ventilation. OBMs were computed using a sliding windows of 30 minutes, from 8 hours before the transition, to 4 hours after it with a shift of five minutes. The median and IQR were computed over 68 transition events. For each of the five category, one biomarker with low p value is represented. OBMs definition are available in Table S1.
